# 

**Published:** 1904-02-13

**Authors:** 


### CONTENTS.

**Doctors and Drug Supply** - - - - - - - 347

**The Psychology of Tuberculosis** - - - - - - 348

**Annotations** - - - - - - - - - - - 349

**Notes and News** - - - - - - - - - - 350

**Hospital Clinics and Medical Progress—**

What We Owe to Experiments on Animals - - - - - 351

PROGRESS IN GYNÆCOLOGY - - - - - - - - 354

PROGRESS OF THE SURGERY OF THE VERMIFORM APPENDIX - 355

PROGRESS IN DISEASES OF THE HEART - - - - - - 356

**New Appliances and Things Medical** - - - 357

**The Book World of Medicine and Science** - - 358

**Hospital Administration—**

Rise and Growth of Vaccination Law.—V. - - - - - 359

British Health Stations.—I. - - - - - - - 361

Glances at the Hospitals - - - - - - - - 361

The Story of the Insane from Year to Year - - - - - 363

**Editor's Letter-Box** - - - - - - - - - 364

**The Student of Medicine** - - - - - - - - 364

*NURSING SECTION.*

**Notes on News from the Nursing World—**

The Nurses' Co-operation—A Strange Lapse of Memory—Nurses and Students—The Midwives Board and the Irish Schools—District Work for Probationers—Overwork at Poplar and Stepney Sick Asylum—Trained Nurses' Annuity Fund—Death of a Matron—Poor Law Nurses and the Midwives Act—St. Catherine's Hospital, Cawnpore—The Appointment of a Superintendent Nurse—The Charges Against a Workhouse Matron—Unwise Advice at Rochdale—The Superintendent of Canterbury Nurses' Institute—The Death of a Probationer at Walsall Union Infirmary—Short Items - - - - - - - - - 265-268

**The Nursing Outlook** - - - - - - - - 269

**Lectures Upon the Nursing of Mental Diseases** 270

**A Nursing Experience on Mount Lebanon** - - 271

**The Central Midwives Board** - - - - - - 272

**The Anglo-American Nursing Home at Rome** - 273

**Everybody's Opinion** - - - - - - - - 273

**Novelties for Nurses** - - - - - - - - - 274

**To Nurses** - - - - - - - - - - - - 274

**Appointments** - - - - - - - - - - - 274

**Where to Go** - - - - - - - - - - - - 274

**Echoes from the Outside World** - - - - - - 275

**A Book and its Story** - - - - - - - - 276

**Notes and Queries** - - - - - - - - - - 277

**The Nurses' Bookshelf** - - - - - - - - 278

**For Reading to the Sick** - - - - - - - - 278

